# Stent-in-stent deployment across the papilla for malignant hilar biliary obstruction using novel slim multi-hole metal stents

**DOI:** 10.1055/a-2714-2453

**Published:** 2025-10-13

**Authors:** Hirotsugu Maruyama, Yuki Ishikawa-Kakiya, Yuji Kawata, Tatsuya Kurokawa, Yoshinori Shimamoto, Kojiro Tanoue, Yasuhiro Fujiwara

**Affiliations:** 1 12935 Gastroenterology, Osaka Metropolitan University Graduate School of Medicine School of Medicine, Osaka, Japan

**Fig. 1:**
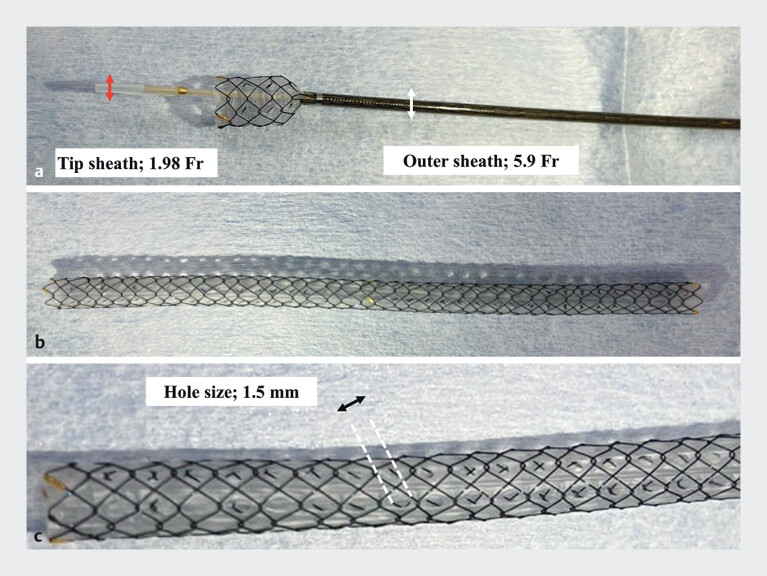
Novel multi-hole metal stent design. **a** The outer sheath has a diameter of 5.9F and the tip is tapered to 1.98F for easier insertion. **b** Structural design of the stent. **c** Each side hole has a diameter of 1.5 mm.

We previously reported stent-in-stent (SIS) placement of multi-hole self-expandable metallic stents (MHSEMS) as an innovative endoscopic technique 1 2 . This method has the advantage of avoiding obstruction of hepatic duct side branches and preventing tumor in-growth. However, because the stent is deployed above the papilla, concerns remain regarding its removal after long-term placement. In addition, passing a second MHSEMS through the holes to the liver side can be technically challenging. We report a method to overcome these two issues using a novel MHSEMS (HANAROSTENT Biliary Multi-Hole Benefit; M.I. Tech Co., Ltd, Pyeongtaek, South Korea) ( Fig. 1 ) with a slim delivery system.

**Fig. 2:**
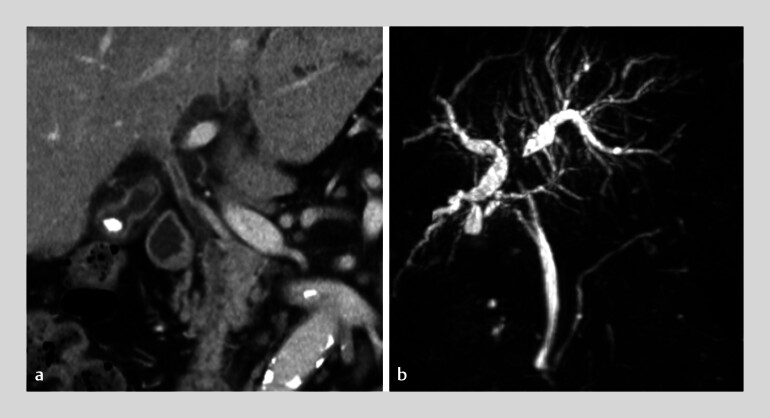
Computed tomography (CT) and magnetic resonance cholangiopancreatography (MRCP) images of malignant hilar biliary obstruction. **a** Contrast-enhanced CT reveals bile duct wall thickening extending from the common hepatic duct to the hepatic hilum. **b** MRCP demonstrates hilar biliary obstruction with upstream intrahepatic bile duct dilatation.

**Fig. 3:**
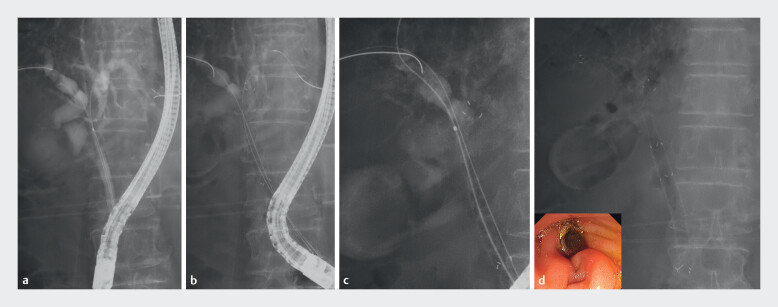
Endoscopic and fluoroscopic images of stent deployment using the novel MHSEMS. **a** Fluoroscopy showing a Bismuth type IV hepatic hilar obstruction. **b** The novel-designed multi-hole metal stent was deployed from the left hepatic duct across the papilla. **c** A guidewire was advanced into the posterior bile duct through a 1.5-mm side hole in the stent lumen. **d** Endoscopic bilateral stent-in-stent deployment across the papilla using MHSEMS with a slim delivery system.

A 76-year-old woman with hilar bile duct cancer presented with jaundice ( Fig. 2 ). An inside plastic stent had been placed, but it was replaced with metal stents because the patient declined cancer treatment ( Video 1 ). First, a 0.025-inch guidewire (GW) was inserted into the left and posterior bile duct, and a Bismuth type IV hepatic hilar obstruction was identified fluoroscopically. MHSEMS (8 mm, 10 cm) was then deployed from the left hepatic duct across the papilla. Next, the GW used for the first stent placement was advanced into the right posterior hepatic bile duct through the 1.5-mm hole within the stent lumen. A second MHSEMS (8 mm, 12 cm) was deployed in the right posterior hepatic bile duct across the papilla ( Fig. 3 ). Passage of the second MHSEMS through the hole was easy, and no adverse events were observed.

Successful stent-in-stent deployment using novel multi-hole metal stents with a slim delivery system to treat malignant hepatic hilar biliary obstruction (M2 SIS).Video 1

This stent features a slim delivery system for easy insertion. Placed across the papilla, its distal end is visible and can be grasped endoscopically for removal. This video also shows a case in which a stent was successfully removed to treat cholangitis resulting from recurrent biliary obstruction.

The SIS technique using this new design MHSEMS reduces technical difficulty, which can be a promising new treatment option.
